# Prevalence of anemia in pregnant women in Styria, Austria—A retrospective analysis of mother-child examinations 2006–2014

**DOI:** 10.1371/journal.pone.0219703

**Published:** 2019-07-25

**Authors:** Sereina Annik Herzog, Gudrun Leikauf, Heidelinde Jakse, Andrea Siebenhofer, Martin Haeusler, Andrea Berghold

**Affiliations:** 1 Institute for Medical Informatics, Statistics and Documentation, Medical University of Graz, Graz, Austria; 2 Mother-child booklet service of the Styrian State Health Insurance Clinic (Steiermärkische Gebietskrankenkasse, Stmk. GKK), Graz, Austria; 3 Institute of General Practice and Evidence-based Health Services Research, Medical University of Graz, Graz, Austria; 4 Institute of General Practice, Goethe-University Frankfurt am Main, Frankfurt am Main, Main, Germany; 5 Department of Obstetrics and Gynecology, Division of Obstetrics and Maternal Fetal Medicine, Medical University of Graz, Graz, Austria; Hawassa University College of Medicine and Health Sciences, ETHIOPIA

## Abstract

**Background:**

Many women suffer from anemia during their pregnancy. Austria, a central European country, has an instituted mandatory prenatal care system and therein two anemia screening tests (before end of week 16 and in weeks 25-28) are scheduled. Epidemiological data on the prevalence of anemia in pregnant women in Austria are missing.

**Methods:**

We analysed data from Styria, an Austrian federal state, to determine the prevalence of anemia diagnosed in pregnant women aged 15-45 years with at least one examination in the first and second time period using the cut-off hemoglobin (Hb) concentration of 11 g/dl as recommended by the World Health Organisation (WHO). Sensitivity analyses for cut-off values with 10.5 and 7 g/dl (severe anemia) were performed. The STROBE checklist was applied for this retrospective cohort study.

**Results:**

The study included anemia screening tests from 25,922 women during 31,429 pregnancies from 2006-2014. Anemia was diagnosed in either time period in 13.7% (95% confidence interval (CI) 13.4-14.1) of pregnancies, in the first time period in 2.2% (95% CI 2.0-2.2), and in the second time period in 13.0% (95% CI 12.6-13.4). The annual age-adjusted anemia prevalence showed no change over time. Reducing the cut-off value to 10.5 g/dl resulted in an anemia prevalence in either time period of 5.6% (95% CI 5.3-5.8). The pattern of a higher prevalence in the second time period remained unchanged. Severe anemia (Hb <7 g/dl) was diagnosed in four pregnancies (0.01%).

**Conclusion:**

The estimated anemia prevalence of around 14% in pregnant women in Styria (Austria) is stable over the observed time window (2006-2014) and almost all are diagnosed in the second test period (in weeks 25-28). It seems that in a developed country like Austria the first examination (before week 16) is not mandatory for pregnancy care. However, in other countries where a high prevalence of anemia is common due to risk factors such as malaria and HIV, early screening in pregnancy might be very important.

## Introduction

Many women around the world, in industrialized as well as in developing countries, suffer from anemia during their pregnancy [1, 2]. The most common form of anemia in pregnant women is the iron deficiency anemia. It is estimated that 50% of anemia is attributable to iron deficiency [1], however, this estimation varies among different populations and areas [1, 3, 4]. During pregnancy, iron deficiency may be associated with multiple adverse outcomes for both mother and infant, e.g. increased risk of maternal and perinatal mortality or low birth weight [3, 5, 6]. For the year 2011, the World Health Organisation (WHO) estimated for pregnant women aged 15-49 years a global anemia prevalence of 38.2% (95% confidence interval (CI) 33.5–42.6), whereas the estimate for the European WHO region was 25.8% (95% CI 19.8–33.6) [1].

Various guidelines exist regarding anemia screening in pregnancy, however, they differ in the degree of recommendation as well as in the number of screening tests as the following two examples illustrate [7]. The 2009 Veterans Affairs/Department of Defense (VA/DoD) Clinical Practice Guideline for management of pregnancy recommends an anemia screening during weeks 6 to 8 and does not recommend a repeated testing for all women [8]. In contrast, the Clinical Guideline by the National Institute for Health and Care Excellence (NICE) recommends a screening test early in pregnancy and again at 28 weeks [9]. In Austria, every pregnant woman is tested twice for anemia as a part of the check-up’s specified in the mother-child booklet (MCB). Therein hemoglobin (Hb) levels are measured once up to the 16th week of pregnancy and a second time between pregnancy week 25 and 28 [10]. Hb levels are used as a proxy for iron status [2] and are therefore used to diagnose anemia. Since 1974, the Austrian government has required certain examinations for mothers and their newborns to be entered into the MCB until the child reaches the age of 62 months [11]. As a result of new developments in research, this program is now under review by the Austrian Ministry of Labour, Social Affairs, Health and Consumer Protection [12]. During a structured assessment of existing guidelines [7] and discussions by a panel of experts, the question arose whether the two blood samples (before week 16 and in weeks 25-28) that are currently required during pregnancy are actually necessary. To our knowledge, no epidemiological data for the prevalence of anemia during pregnancy are published for Austria.

Therefore, the main aim of this study was to determine the prevalence of anemia diagnosed in pregnant women in Styria, a federal state in Austria, covering years 2006-2014. Lab results from pregnant women containing Hb values from both the first and from the second time period were examined.

## Materials and methods

This is a retrospective cohort study where all data were pseudonymized before access. The study was approved by the Ethics Committee of the Medical University of Graz; Austria (EK Nr.: 28-489 ex 15/16). Reporting of this research adheres to the STROBE (STrengthening the Reporting of OBservational studies in Epidemiology) guidelines; see S1 Appendix [13].

The analysis was based on results of measured Hb levels done by the MCB service in the period from January 1, 2006 through December 31, 2014 that were exported from the laboratory data system of Styrian Health Insurance (Steiermärkische Gebietskrankenkasse or Stmk. GKK), Austria. Identifying fields that fell under data-privacy laws were pseudonymized. The dataset was restricted to examinations with no missing information about Hb level and month and year of birth, and age between 15-45 years. The routinely collected data contains no information on further parameters such as intake of supplements during pregnancy, smoking status, or altitude.

### Hemoglobin level measurement and anemia

The Hb levels were measured by Sysmex XS1000i for the whole study period. For the analysis, anemia was defined as an Hb level <11g /dl and for severe anemia <7 g/dl which is in accordance with the WHO definition [3].

### Defining pregnancies

We had information about week or month of gestation for the examination and a unique ID for each woman but no further information which examinations belong to the same pregnancy. Similar to another retrospective analysis of mother-child examinations, we used an algorithm to specify which examination belong most likely to the same pregnancy. Therefore, we calculated for each examination the potential time window of the pregnancy, i.e. from date of examination −7 * (week of gestation + 1) to date of examination +7 * (42 − week of gestation) [14]. At each examination, we checked which other examinations fell into the probable time window of the pregnancy. Examinations without information about the week of gestation fell either into the time window of pregnancy from another examination or otherwise were defined as being a separate pregnancy. If there was no information for several examinations in sequence, we classified them as belonging to the same pregnancy when they were performed within a time window of 300 days. Examinations were classified as belonging to the same pregnancy when the information on the probable time window of the pregnancy from the different examinations was not contradictory. If it was, we excluded the woman and all her examinations from the data set. Women with no information at all about week or month of gestation for all their examinations were excluded from the data set as well. We also excluded pregnancies with only one examination or with missing information on week or month of gestation, i.e. full information on week or month of gestation for all examinations in a pregnancy with at least two examinations was required.

### Statistical analysis

For the main analysis, we selected pregnancies for which we had ≥2 examinations with at least one in the first time period (week of gestation ≤16) and at least one examination in the second time period (25≤ week of gestation ≤28). The two time periods defined for the main analysis reflect the recommendation in the MCB. If several examinations were available in a time period, then the earliest examination was taken to investigate the presence of anemia. Anemia was defined as a Hb level <11 g/dl (see section ‘Hemoglobin level measurement and anemia’).

We analyzed the change in Hb level between first and second time period using a linear mixed model with a random intercept. The overall anemia prevalence was estimated by dividing the number of pregnancies with anemia diagnosis by the total number of pregnancies. The corresponding 95% confidence intervals (95% CI) were calculated based on the exact method under a binomial distribution. Annual anemia prevalence estimations are age-adjusted. Age adjustment was done using direct standardization with the 2006 female census population of Styria as reference population and using 5-year age groups from 15-45. Changes in anemia prevalence over time were analyzed with a logistic regression model; trend in time was assessed by interpreting the corresponding z-statistic for the linear predictor time.

We conducted for the overall anemia prevalence and the annual age-adjusted prevalence two sensitivity analyses. First, we extended the second time window to range from 24 to 30 weeks of gestation because a substantial amount of examinations took place just outside the second time period. Second, we kept the original time periods but we investigated what happens to the anemia prevalence if we changed the cut-off to <10.5 g/dl and <7 g/dl (severe anemia). The cut-off of 10.5 g/dl is mentioned, e.g. in the Clinical Guidelines by NICE or by the Center of Disease Control and Prevention (CDC), as a threshold to diagnose anemia in pregnant women [9, 15]. A p-value <0.05 was considered statistically significant. All analyses were performed using the R statistical software (version 3.5.2) [16].

## Results

**Fig 1:**
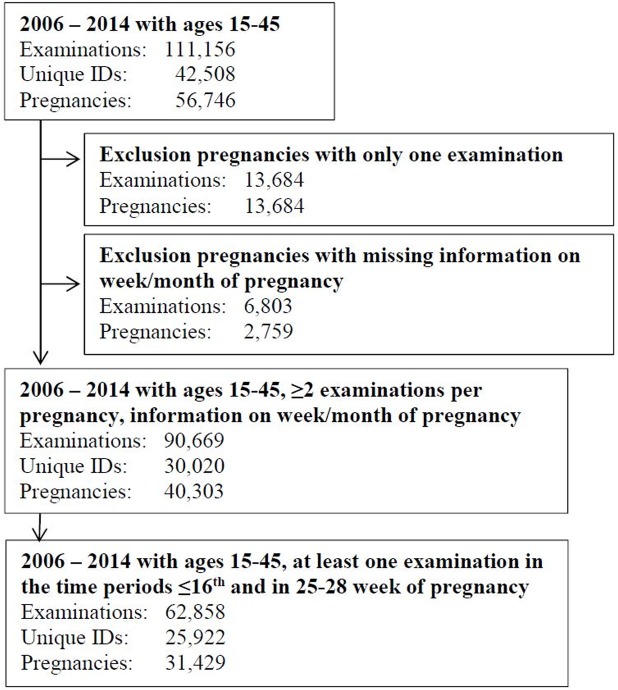
Flowchart explaining the selection of data for the analysis of prevalence of anemia, motherchild examinations, Styria, Austria, 2006–2014. Unique ID = each woman had a unique code assigned, so that examinations could be identified as pertaining to that particular woman; Hb = hemoglobin.

For the study period of 2006-2014, there were 111,156 anemia screening tests. After application of the inclusion and exclusion criteria, there were 90,669 screening tests from 30,020 women and their 40,303 pregnancies (Fig 1). The median age in all pregnancies (first examination) was 29.1 years (interquartile range (IQR) 25.1-33.0) in 2006 and 29.8 years (IQR 26.0-33.4) in 2014. Further 8,874 pregnancies were excluded from the main analysis since there was not at least one examination in each of the time periods specified. For the main analysis, 31,429 pregnancies (78.0%) were included.

### Prevalence of anemia in pregnant women

**Fig 2:**
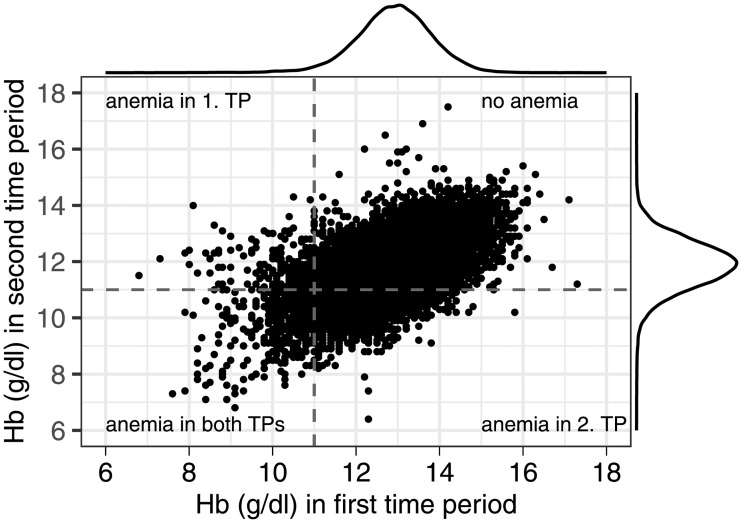
Hemoglobin levels (g/dl) in the first time period and second time period. Hemoglobin levels (g/dl) in the first time period (week of gestation ≤16; median 12.9 g/dl, range 6.8-17.3) and second time period (25≤week of gestation≤28; median 11.9 g/dl, range 3.9-17.5). Marginal density is shown for the two different time periods. Anemia is indicated using the cut-off <11 g/dl. Hb = hemoglobin; TP = time period.

Fig 2 shows the measured Hb levels in the first and second time period; indicating a shift towards lower values later in pregnancy. The weekly decline in Hb was estimated as 0.06 g/dl (linear mixed model, p<0.001).

**Fig 3:**
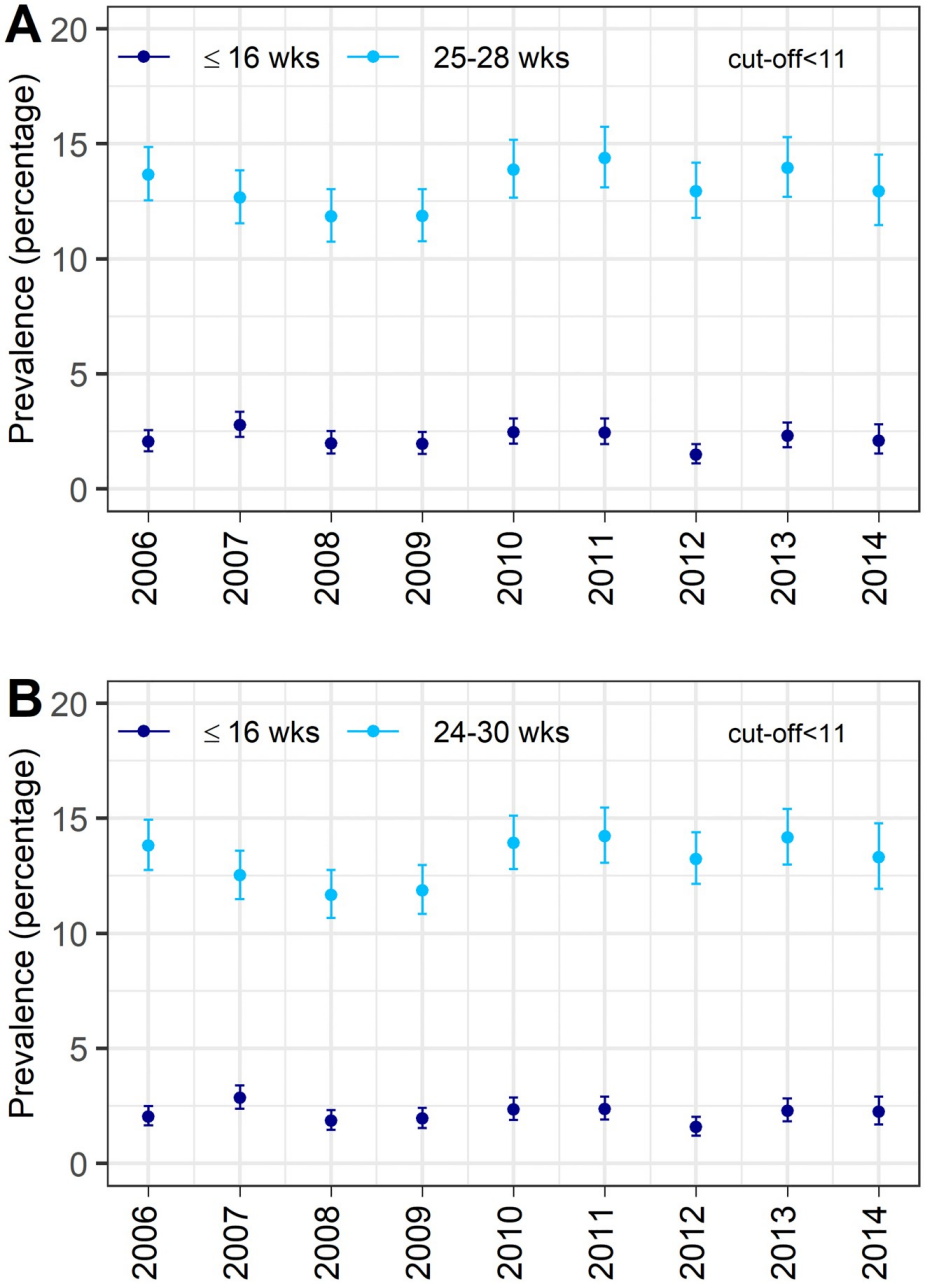
Annual age-adjusted anemia prevalence, ages 15-45 (cut-off <11 g/dl). Annual age-adjusted anemia prevalence with cut-off Hb <11 g/dl in the first time period (dark blue) and in the second time period (light blue) with the corresponding 95% CI. Age adjustment was done using direct standardisation with the 2006 female census population of Styria as reference population and using 5-year age groups from 15-45. Panel A: first time period with week of gestation ≤16, second time period with 25≤week of gestation≤28. Panel B: first time period with week of gestation ≤16, second time period with 24≤week of gestation≤30. Hb = hemoglobin; wks = weeks, CI = confidence intervals.

Anemia was diagnosed in either time period in 13.7% (4,319/31,429; 95% CI 13.4-14.1) of pregnancies, in the first time period in 2.2% (679/31,429; 95% CI 2.0-2.2), and in the second time period in 13.0% (4,086/31,429; 95% CI 12.6-13.4) using the cut-off <11 g/dl. Fig 3A shows the annual age-adjusted anemia prevalence for anemia. No change over time is observed for the age-adjusted anemia prevalence (linear test of trend: first period p = 0.330, second period p = 0.191).

For the sensitivity analysis with the extended second time period from week 24 to week 30, there were 36,728 pregnancies (91.1%) from 29,600 women who had at least one examination in each time period. Anemia was diagnosed in 13.8% (5,075/36,728; 95% CI 13.5-14.2) of the pregnancies in either time period, in the first time period in 2.1% (787/36,728; 95% CI 2.0-2.3) and in the second time period in 13.1% (4,795/36,728; 95% CI 12.7-13.4) using the cut-off <11 g/dl. Fig 3B shows the annual age-adjusted anemia prevalence for anemia. No change over time was observed for the age-adjusted anemia prevalence in the first time period (linear test of trend: p = 0.461), however, there was a small increase for the anemia prevalence diagnosed in the second time period (linear test of trend: increase in prevalence per year of 0.14%, 95% CI 0.003% to 0.277%, p = 0.048).

### Influence of changing cut-off on anemia prevalence in pregnant women

**Fig 4:**
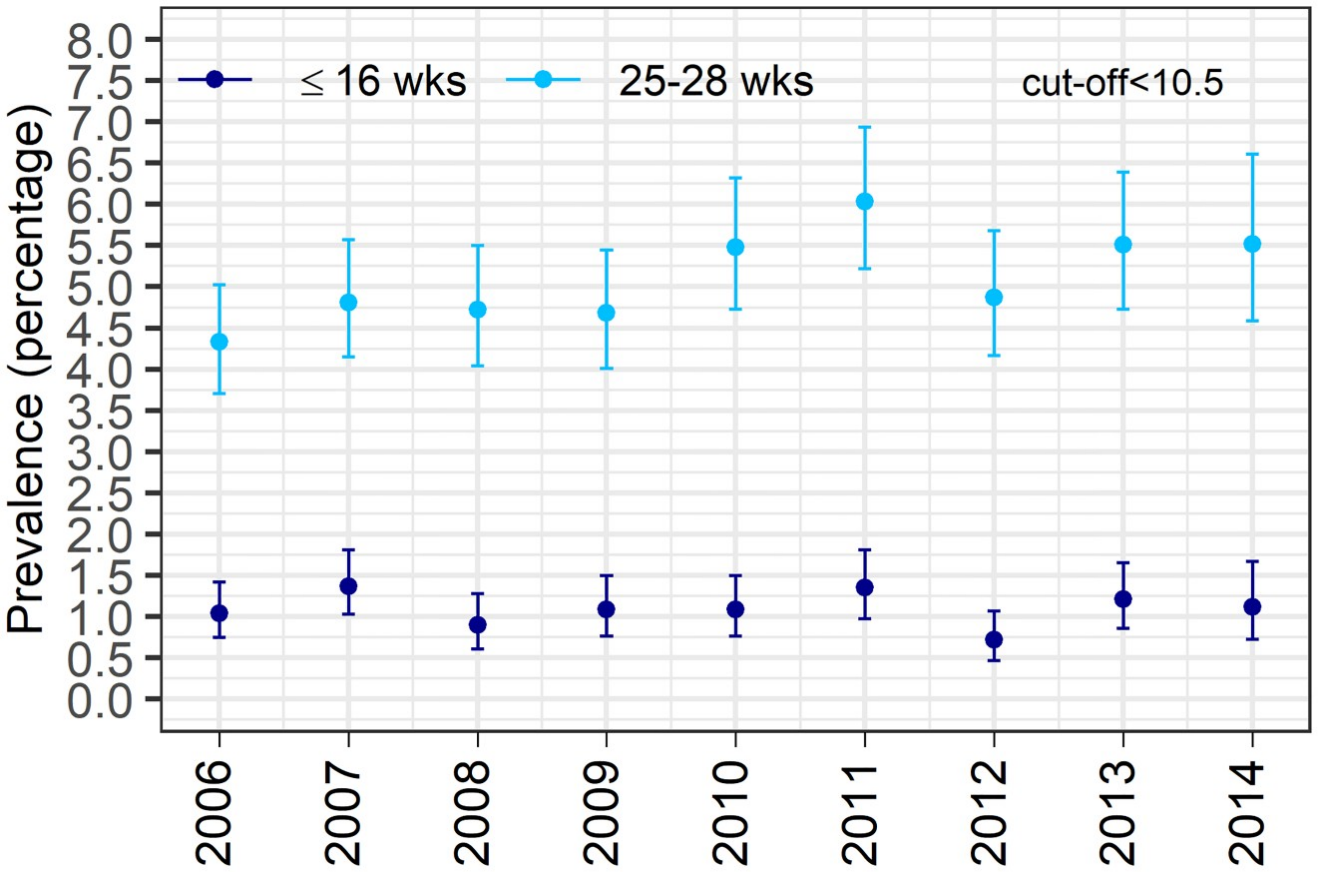
Annual age-adjusted anemia prevalence, ages 15-45 (cut-off <10.5 g/dl). Annual age-adjusted anemia prevalence in the first time window (week of gestation≤16, dark blue) and in the second time window (25≤week of gestation≤28, light blue) with the corresponding 95% CI for anemia being defined as Hb level <10.5 g/dl. Age adjustment was done using direct standardisation with the 2006 female census population of Styria as reference population and using 5-year age groups from 15-45. Hb = hemoglobin, wks = weeks, CI = confidence intervals.

Changing the cut-off from <11 g/dl to <10.5 g/dl to define anemia, it was diagnosed in 5.6% (1743/31,429; 95% CI 5.3-5.8) of the pregnancies in either time period, in the first time period 1.1% (339/31,429; 95% CI 1.0-1.2) and in the second time period in 5.0% (1,578/31,429; 95% CI 4.8-5.3). Fig 4 shows the annual age-adjusted anemia prevalence. There was a small increase for the anemia prevalence diagnosed in the first time period (linear test of trend: increase in prevalence per year of 0.14%, 95% CI 0.042% to 0.238%, p = 0.008) as well as for the second time period (linear test of trend: increase in prevalence per year of 0.15%, 95% CI 0.052% to 0.248%, p = 0.002).

Severe anemia (<7 g/dl) was diagnosed in only four pregnancies throughout the whole study period 2006–2014, i.e. a prevalence of 0.01% severe anemia in either time period. One case was diagnosed before week 16 and 3 cases were seen between week 25 and 28.

## Discussion

For the study period of 2006-2014, we found an anemia prevalence for pregnant women aged 15-45 years in Styria, Austria, of 13.7% (95% CI 13.4-14.1), using the cut-off <11 g/dl for the Hb level. The annual age-adjusted anemia prevalence showed no change over time for the two time periods (up to the 16th week, between week 25 and 28). Extending the second time period from 24th to 30th week of pregnancy did not change the prevalence estimations, however, there was a small increase over the study period for the anemia prevalence diagnosed in the second time period. If we changed the cut-off to <10.5 g/dl to define anemia, this was diagnosed in 5.6% (95% CI 5.3-5.8) of the pregnancies. Severe anemia (Hb <7 g/dl) was diagnosed in four pregnancies (0.01%) throughout the whole study period. The estimated decline in Hb of 0.06 g/dl per gestation week is in line with a recently published estimation from laboratories in Berlin (Germany) with a reduction of 0.07 g/dl per week [17].

According to WHO definition, the anemia prevalence of 13.7% (95% CI 13.4-14.1) in pregnant women indicates it to be a mild public health problem in Styria, Austria [3]. For the year 2011, the WHO estimated an anemia prevalence of 25% (95% CI 13-45) for pregnant women aged 15-49 in Austria [1] which is almost twice as much as shown by our data. However, Austria is indicated in this report as a country that does not have national or subnational data on anemia prevalence. The WHO estimation for Austria came from a model which used information from countries in the same region [1, 4]. On the other hand, the WHO reported for the period 1993-2005 for Austria a regression based anemia prevalence estimation of 15.5% (95% CI 3.8-45.6), i.e. also no survey data were available [18]. This estimation is very similar to ours, however, the two study periods do not overlap.

In 2012, the World Health Assembly Resolution defined, among other things, the target to reduce anemia in women of reproductive age by 50% until 2025 [19]. In our case it seems to be a mild public health problem which makes it more difficult to reduce it even further. In the study period 2006-2014 we observed no change over time in anemia prevalence whereas an estimated global fall of anemia prevalence in pregnant women, from 43% in 1995 to 38% in 2011 was seen [19].

Anemia is often diagnosed by measuring blood Hb concentration as an inexpensive screening tool, serving as a proxy for iron status [2]. For the main analysis in this study the WHO standard definition of Hb <11 g/dl for pregnant woman was used [3]. It is well known, that Hb concentration alone does not determine the cause of anemia [1] and diagnoses only the most severe forms of iron deficiency, i.e. anemia [17]. Hb can still be inconspicuous, while serum ferritin levels are already reduced for iron deficiency and iron-deficient erythropoiesis [17]. Screening by serum ferritin measurements additionally to Hb would be beneficial but would have to bear comparison with cost-benefit calculations [3, 17, 20].

One of the limitations of our study is the retrospective study design. In our dataset no information was available on further parameters such as iron intake, other medications, or medical problems [21]. However, for this study an extensive data collection from a laboratory specialized in screening examinations (preventive medical examinations) for pregnant women covering a long time period 2006-2014 was available. The data are only from one federal state of Austria, Styria, covering 14.3% (2014) of the Austrian population and therefore should reflect the situation in Austria well as the examination schedule recommended by the MCB is the same in whole Austria. Despite these limitations, we are able to provide for the first time data-based estimates of anemia in pregnant women for one federal state in Austria (Styria).

## Conclusion

The estimated anemia prevalence of around 14% in pregnant women in Styria (Austria) is stable over the observed time window (2006-2014) and almost all are diagnosed in the second test period (in weeks 25-28). It seems that in a developed country like Austria the first examination (before week 16) is not mandatory for pregnancy care. However, in other countries where a high prevalence of anemia is common due to risk factors such as malaria and HIV, early screening in pregnancy might be very important [22, 23]. Furthermore, our study indicates that prevalence estimates based solely on data from surrounding countries because of a lack of national data (WHO report for 2011 [1]), might be misleading for public health authorities; e.g. in Austria the classification of anemia prevalence in pregnant women has to be changed from moderate (25%—WHO estimate) to mild (14%—our study) [3].

## Supporting information

S1 Appendix STROBE—Checklist for cohort studies. (PDF)
